# Revealing the roles of TLR7, a nucleic acid sensor for COVID-19 in pan-cancer

**DOI:** 10.1016/j.bsheal.2023.05.004

**Published:** 2023-05-09

**Authors:** Zhijian Huang, Yaoxin Gao, Yuanyuan Han, Jingwen Yang, Can Yang, Shixiong Li, Decong Zhou, Qiuyan Huang, Jialiang Yang

**Affiliations:** a Department of Breast Surgical Oncology, Clinical Oncology School of Fujian Medical University, Fujian Cancer Hospital, Fuzhou 350014, China; b Biotherapy Center, The First Affiliated Hospital of Zhengzhou University, Zhengzhou 450052, China; c Institute of Medical Biology, Chinese Academy of Medical Sciences and Peking Union Medical College, Kunming 650000, China; d Department of Clinical Pharmacy, Clinical Oncology School of Fujian Medical University, Fujian Cancer Hospital, Fuzhou 350014, China; e Geriatric Hospital of Hainan Medical Education Department, Haikou 571100, China; f Geneis Beijing Co., Ltd, Beijing 100102, China; g Qingdao Geneis Institute of Big Data Mining and Precision Medicine, Qingdao 266000, China

**Keywords:** COVID-19, TLR7, Pan-cancer, SARS-CoV-2, Prognosis

## Abstract

Recent studies suggested that cancer was a risk factor for coronavirus disease 2019 (COVID-19). Toll-like receptor 7 (TLR7), a severe acute respiratory syndrome 2 (SARS-CoV-2) virus's nucleic acid sensor, was discovered to be aberrantly expressed in many types of cancers. However, its expression pattern across cancers and association with COVID-19 has not been systematically studied. In this study, we proposed a computational framework to comprehensively study the roles of TLR7 in COVID-19 and pan-cancers at genetic, gene expression, protein, epigenetic, and single-cell levels. We found TLR7 mRNA expression was significantly up-regulated in 6 cancer types and down-regulated in 6 cancer types, further validated in the HPA database at the protein level. The genes significantly co-expressed with TLR7 were mainly enriched in the toll-like receptor signaling pathway, endolysosome, and signaling pattern recognition receptor activity. In addition, the abnormal TLR7 expression was associated with Mismatch repair (MMR), microsatellite instability (MSI), tumor mutational burden (TMB) in various cancers. Mined by the ESTIMATE algorithm, the expression of TLR7 was also closely linked to various immune infiltration patterns in pan-cancer, and TLR7 was mainly enriched in macrophages, as revealed by single-cell RNA sequencing. Finally, TLR7 expressions were very sensitive to a few targeted drugs, such as Alectinib and Imiquimod. In conclusion, TLR7 might be essential in the pathogenesis of COVID-19 and cancers.

## Highlights

• Scientific question: Toll-like receptor 7 (TLR7), a severe acute respiratory syndrome 2 (SARS-CoV-2) virus’s nucleic acid sensor, was discovered to be aberrantly expressed in many types of cancers. However, its expression pattern across cancers and association with coronavirus disease 2019 (COVID-19) has not been systematically studied.
• Evidence before this study: COVID-19 was a disease mainly characterized by damage to the respiratory system caused SARS-CoV-2. Recent studies suggested that cancer was a risk factor for COVID-19.
• New findings: In this study, we proposed a computational framework to comprehensively study the roles of TLR7 in COVID-19 and pan-cancers at genetic, gene expression, protein, epigenetic, and single-cell levels. As a result, TLR7 expression was found to be higher in the lung of mice infected with SARS-CoV-2 than that in the control group.
• Significance of the study: In this study, revealing the roles of TLR7, a nucleic acid sensor for COVID-19 in pan-cancer. These findings might be necessary for preventing SARS-CoV-2 infection and alleviating cytokine storms in infected cancer patients.

## 1 Introduction

Coronavirus disease 2019 (COVID-19) was a disease mainly characterized by damage to the respiratory system caused by severe acute respiratory syndrome coronavirus 2 (SARS-CoV-2) [1]. According to the survey, most SARS-CoV-2 patients had mild to moderate symptoms, but 15% of them developed severe pneumonia, and 5% eventually developed acute respiratory distress syndrome (ARDS) and multiple organ dysfunction [2], [3]. Cancer patients needed extensive care because they experienced more severe issues with SARS-CoV-2 during the COVID-19 pandemic [4], [5]. Therefore, to keep treating cancer patients safely throughout the COVID-19 pandemic, it would be helpful to investigate SARS-CoV-2 control strategies in cancer patients [4].

Toll-like receptor 7 (TLR7) was a TLR family and a gene-encoded protein essential for pathogen recognition and innate immune reaction. According to recent reports [6], the pathophysiology of the Middle East respiratory disease (MERS) and the severe acute respiratory syndrome (SARS-CoV) were closely related to toll-like receptors [7]. The GG (homozygous dominant genotype) form of the TLR7 single nucleotide polymorphisms (SNP) may be a genetic risk factor for COVID-19 infection, severe disease, and poor clinical outcomes because it is a natural ligand for single-stranded RNA viruses [7]. According to studies, common RNA viruses like SARS-CoV-2, which caused COVID-19, infect the innate immune system by binding to TLRs 3, 7, and 8 [8]. TLR7 and TLR8 were phylogenetically and structurally very similar. In addition to recognizing GU-rich sequences in single-stranded RNA from viruses [9], [10], they also took part in various intracellular signaling cascades that eventually resulted in the expression of factors and chemokines by proinflammatory cells [11].

MyD88, an adaptor protein that activated downstream NF-κB driver genes [12], was also connected to TLR7/8. According to a recent study, men under 60 with the lethal COVID-19 disease had more pronounced TLR7 variants than controls [13]. A potential new target for COVID-19 treatment was TLR7, a critical component of COVID-19 infection and progression [9].

In addition to COVID-19, multiple tumors' tumorigenesis has been reported to involve TLR7, and treating infectious diseases and cancer with immunotherapy has become a potent clinical strategy. For example, Resimod (R848), a TLR7 agonist, was effective in treating several cancers, including breast, pancreatic, and colorectal cancer [14], [15], and alum adjuvant and built-in TLR7a work together to enhance MUC1 glycopeptide-specific memory CD8+ T cell immunity [16]. Furthermore, co-immobilization of gold nanoparticles with TLR7 ligand derivatives, i.e., synthesis of small molecule TLR7 ligand (1V209) and lipoic acid (TA) via 4-, 7-, 10-trioxa-1,13-tridecanediamine and α-mannan Sugar (1V209-αMan-GNPs: sugar nano-adjuvant) significantly enhanced immune stimulation [17]. In conclusion, TLR7 was a potential tumor biomarker that might help cancer patients during the COVID-19 pandemic by acting as a tumor and COVID-19 biomodulator [14]. However, the prognosis of patients with COVID-19 cancer, TLR7 expression, and its relationship to immune infiltration and pan-cancer clinical relevance were still unknown and warrant further investigation.

Single-cell transcriptome sequencing was a new technology for high-throughput transcriptome sequencing at the single-cell level, which could effectively reveal cellular heterogeneity that was masked by bulk RNA-seq methods [18], and help to discover new rare cell types, and further understand the expression regulation mechanism in the process of cell growth. We further investigated TLR7 expression analysis in single-cell.

This study used multiple databases to analyze the expression and prognosis of TLR7 in pan-cancer. In addition, to investigate the potential role of TLR7 in tumor patients by investigating the relationship between TLR7 and immune cell infiltration in specific tumor patients, TLR7 promoter methylation profiles were also collected in the UALCAN database at the same time, and the changes in TLR7 in post-infection animals were validated using the GEO dataset. These findings might be necessary for preventing SARS-CoV-2 infection and alleviating cytokine storms in infected cancer patients [14].

## 2 Materials and methods

### 2.1 Transcriptome data analysis

The Cancer Genome Atlas (TCGA) database (https://portal.gdc.cancer.gov/) [19], the Genotype-Tissue Expression (GTEx) database (https://genome.ucsc.edu/gtex.html), the Gene Expression Omnibus (GEO) database (https://www.ncbi.nlm.nih.gov/geo/), and the Broad Institute Cancer Cell Lines Encyclopedia (CCLE) database (https://sites.broadinstitute.org/ccle) was used to obtain gene expression profiles. mRNA data in normal tissues were obtained from the GTEx project, which included 31 tissues. A distributional analysis of cancer cell line expression levels in 21 tissues was performed, followed by Kruskal-Wallis tests on adjacent tumor tissues and mRNA data from tumor tissues to determine differences between the two. TLR7 expression levels were compared between normal tissue and tumor tissue, as well as between adjacent tumor tissue and tumor tissue. The Wilcox test was used to calculate the significance of differences with a p-value threshold of 0.05. Furthermore, the protein expression of TLR7 was identified using immunofluorescence staining based on the Human Protein Atlas (HPA) (https://www.proteinatlas.org/) database.

### 2.2 Construction and enrichment analysis of gene-gene and protein–protein, gene-disease networks

Open-targets database (https://www.opentargets.org) was used to built TLR7-related disease network. GeneMANIA (https://genemania.org/) [20] was used to construct the gene-gene interaction network; STRING database (https://string-db.org/) was used to construct the protein–protein interaction (PPI) network. Gene Ontology (GO) terminology, the Kyoto Encyclopedia of Genes and Genomes (KEGG), and GSEA were used to examine the intersection mentioned above genes. The term “GO” refers to molecular function (MF), cellular component (CC), and biological process (BP). The “ClusterProfiler” [21] package performed GO, KEGG analysis, and GSEA.

### 2.3 Single-cell analysis of TLR7 expression

Target gene protein expression in different tumors was analyzed by the Clinical Proteomic Tumor Analysis Consortium (https://pdc.cancer.gov/pdc/browse). Moreover, TLR7 single-cell analysis was performed through the PanglaoDB database (https://panglaodb.se/).

### 2.4 Epigenetic methylation analysis and association analysis of methyltransferases

As a form of DNA chemical modification, DNA methylation controls gene expression by altering epigenetics without altering the DNA sequence. To analyze the methylation levels of tumors and normal tissues, we obtained them from the methylation module of the UALCAN database [22]. Subsequently, the correlation between TLR7 expression and the expression of the four methyltransferases were further analyzed. Data visualization via the “ggplot” package Correlation was considered significant and positive when *P* < 0.05 and R > 0.20.

### 2.5 Analysis of tumor mutational burden and genomic alterations in pan-cancer

The total number of substitutions, insertions, and deletions per megabase in the exon coding region of tumor genes was used to calculate tumor mutational burden (TMB) [23]. We obtained pan-cancer MAF files from the TCGA database and calibrated them by dividing the exon region size by the TMB. TLR7 expression was then correlated with TMB using Spearman analysis. Genetic alterations in TLR7 in the TCGA dataset [24] were analyzed through the cBioPortal resource (https://www.cbioportal.org/). The gene changes, and mutation sites of TLR7 were obtained in the “Oncoprint,” “Cancer Type Summary,” and “Mutations” sub-modules.

### 2.6 Analysis of TLR7 expression and DNA mismatch repair system and microsatellite instability

Mismatch repair (MMR) gene expression levels were determined using TCGA expression profiling data. Using the R packages “reshape2″ and ”RColorBrewer,“ the results were visualized as heatmaps. Microsatellite instability (MSI) scores were computed using TCGA Pan-Cancer Mutation Data (https://tcga.xenahubs.net) [25]. The correlation between TLR7 expression and MSI was demonstrated using Spearman's coefficient. The ”fmsb“ package was used to visualize radar charts.

### 2.7 Correlation analysis of TLR7 expression in the immune microenvironment

The tumour microenvironment (TME) was the microenvironment in which tumor cells develop and survive. It comprised several components, including stromal cells, immune cells surrounding tumor cells, and tumor cells. The number of stromal and immune cells in the tumor microenvironment influenced cancer cell growth and development. The expression data (ESTIMATE) algorithm was used to calculate the immune score, stromal score, and ESTIMATE score in malignant tumors by the R package “ESTIMATE”[26].

### 2.8 Analysis of immune checkpoint genes and immune neoantigens

Biological phenomena such as gene fusion, deletion mutation, and point mutation were called neoantigens encoded by mutated genes in tumor cells. Binding affinity scores were calculated for epitopes with a defined length of 8–11 amino acids, and epitopes with scores <500 nm were reported as neoantigens. We then ranked the predicted neoantigens according to the antigenicity index value, affinity, and variant allele frequency. A scanner was used to count the number of neoantigens in each tumor sample and to analyze the relationship between TLR7 expression and the number of antigens. The expression relationship between common immune checkpoint genes and TLR7 was further investigated, and these immune checkpoint genes were extracted and correlated with TLR7 expression.

### 2.9 Clinical correlation analysis

Univariate COX regression analysis on overall survival was performed to determine whether TLR7 expression levels were associated with overall survival (OS) in various cancers. The samples were divided into two groups based on the median of TLR7 expression levels: high and low. The log-rank test was used to determine the significance of differences in survival. TLR7′s diagnostic value in various cancers was estimated using receiver operating characteristic (ROC) analysis methods. The area under the curve (AUC) value was calculated, and the higher the AUC value, the higher the diagnostic value. AUC values of 0.5–0.7, 0.7–0.9, and 0.9–1.0 indicated low, medium, and high prediction effects.

### 2.10 Drug sensitivity of TLR7 in pan-cancer

To investigate TLR7 drug sensitivity in pan-cancer patients, the CallMinerTM database (https://discover.nci.nih.gov/cellminer) was used to obtain NCI-60 compound activity data and RNA-seq expression profiles. To analyze and choose FDA-approved or clinical trial-approved drugs, the R packages “impute,” “limma,” “ggplot2″, and ”ggpubr“ were used for the analysis [26].

### 2.11 Statistical analysis

The Wilcoxon rank sum test and the Spearman rank test were used to investigating expression differences and correlations between the two groups; The hazard ratio was calculated using a Cox proportional hazards regression model (HR). R software version 4.1.2 was used for statistical analysis. *P*-values were used to determine statistics significance (* *P <* 0.05, ** *P <* 0.01, *** *P <* 0.001).

## 3 Results

### 3.1 TLR7 was associated with COVID-19 based on the GEO dataset and Opentargets analysis

**Fig. 1:**
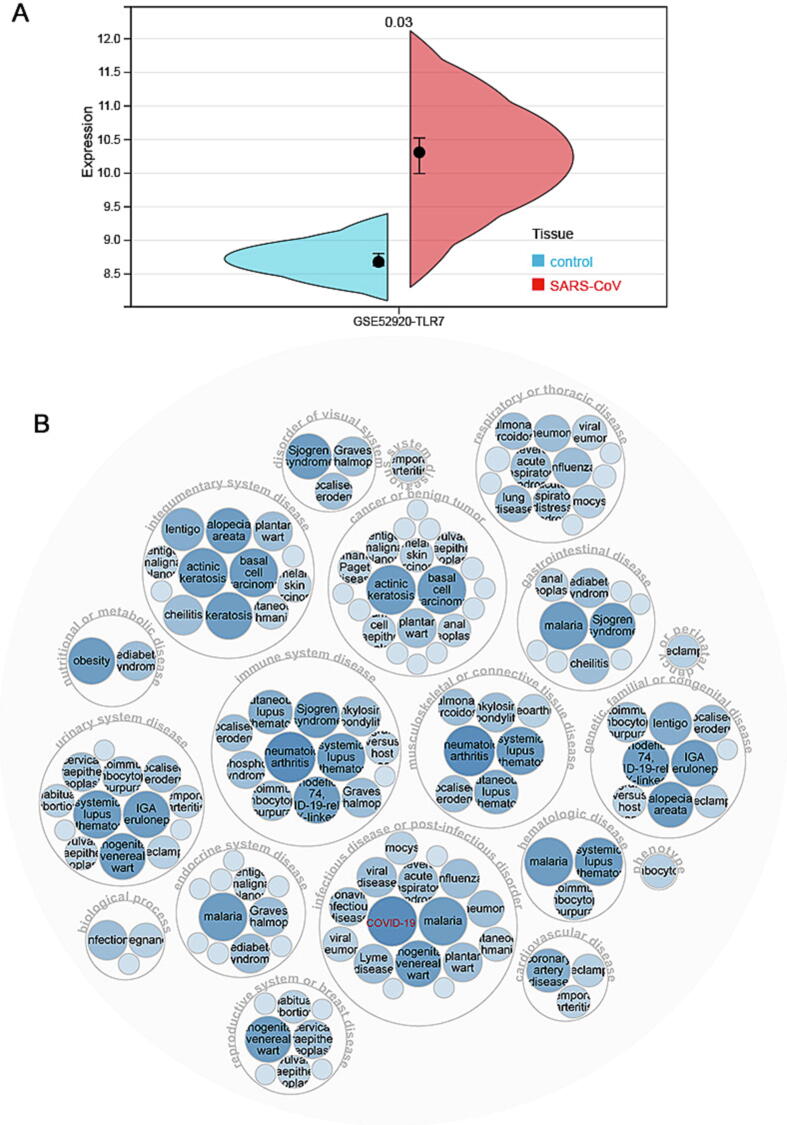
Correlation between TLR7 and COVID-19. A) Changes of TLR7 expression in the lungs of mice infected with SARS-CoV (GSE52920); B) TLR7-related disease network chart using Opentargets. Abbreviations: TLR7, Toll-like receptor 7; COVID-19, coronavirus disease 2019; SARS-CoV, severe acute respiratory syndrome.

It was worthwhile to investigate TLR7 changes in tumors following SARS-CoV-2 infection. Because of the high homology between SARS-CoV-2 and SARS-CoV [22], it was currently thought that changes in TLR7 expression after SARS-CoV infection of cells or animals could be used as a reference for SARS-CoV-2. Therefore, the GSE52920 database was analyzed for changes in TLR7 expression in SARS-CoV- infected mice. TLR7 expression was found to be higher in the lungs of mice compared to the control group (Fig. 1A). This study indicated that TLR7 expression might be elevated following COVID-19 infection. After the intersection with TLR7-related disease using Opentargets, we discovered that TLR7 was associated with COVID-19 (Fig. 1B).

### 3.2 TLR7 was differentially expressed between cancer tissue/cells and normal tissue at mRNA and protein levels

**Fig. 2:**
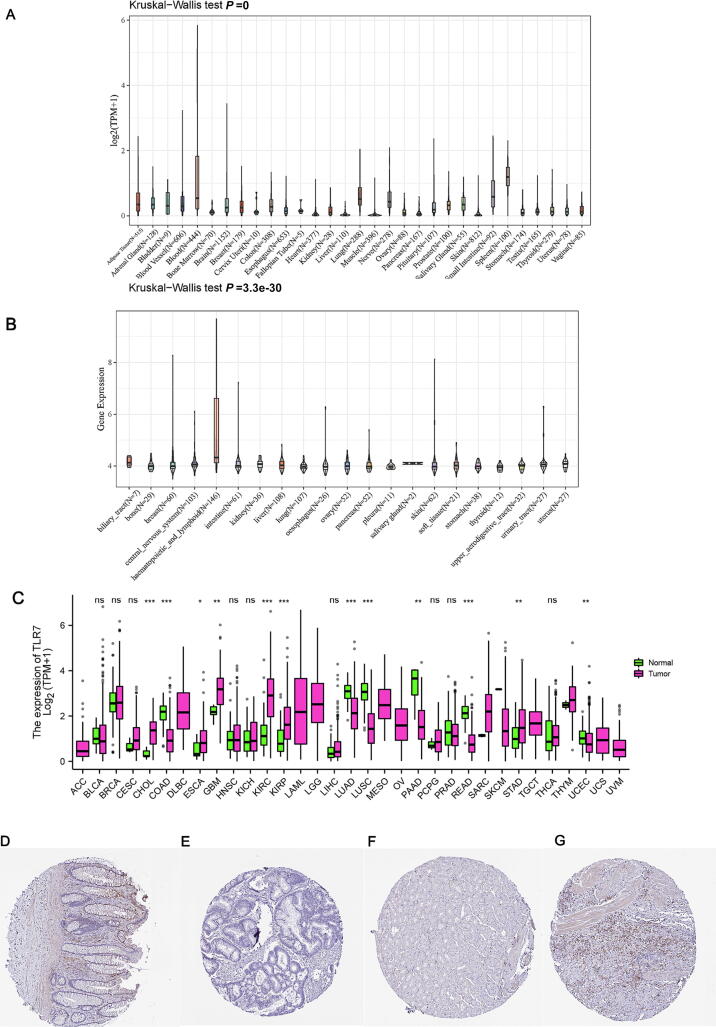
Differential expression analysis of TLR7 in pan-cancer tissues/cells and normal tissues. A) TLR7 mRNA expression levels in normal tissues; B) TLR7 mRNA expression levels in different cancer cell lines in the CCLE database; C) TLR7 mRNA expression levels between cancer and normal tissues in the TCGA database; D) Immunohistochemistry of normal rectum tissue. E) Immunohistochemistry of rectum cancer tissue; F) Immunohistochemistry of normal stomach tissue; G) Immunohistochemistry of stomach cancer tissue. * *P* < 0.05, ** *P* < 0.01, *** *P* < 0.001. Abbreviations: TLR7, Toll-like receptor 7; CCLE, Cancer Cell Line Encyclopedia; TCGA, The Cancer Genome Atlas.

This study looked into the role of human TLR7 expression in pan-cancer. We used the GTEx database to determine the expression pattern of TLR7 in different organ tissues, and we used the CCLE database to determine the expression pattern of TLR7 in 21 different human cancer cell lines. TLR7 was significantly differentially expressed among normal tissues (Fig. 2A) and different cancer cell lines (Fig. 2B). The TCGA database was then used to examine differences in TLR7 expression levels between tumor and normal tissues, revealing the specificity of TLR7 expression (Fig. 2C). TLR7 was highly expressed in CHOL, ESCA, GBM, KIRC, KIRP, and STAD and low in COAD, LUAD, USC, PAAD, READ, and UCEC compared to normal tissues. In addition, we also investigated the immunohistochemistry data of the HPA database to assess the protein level of TLR7. TLR7 was lowly expressed in rectum cancer tissue and highly expressed in stomach cancer tissue at protein levels (Fig. 2D-G).

### 3.3 TLR7 expression analysis in a single cell

**Fig. 3:**
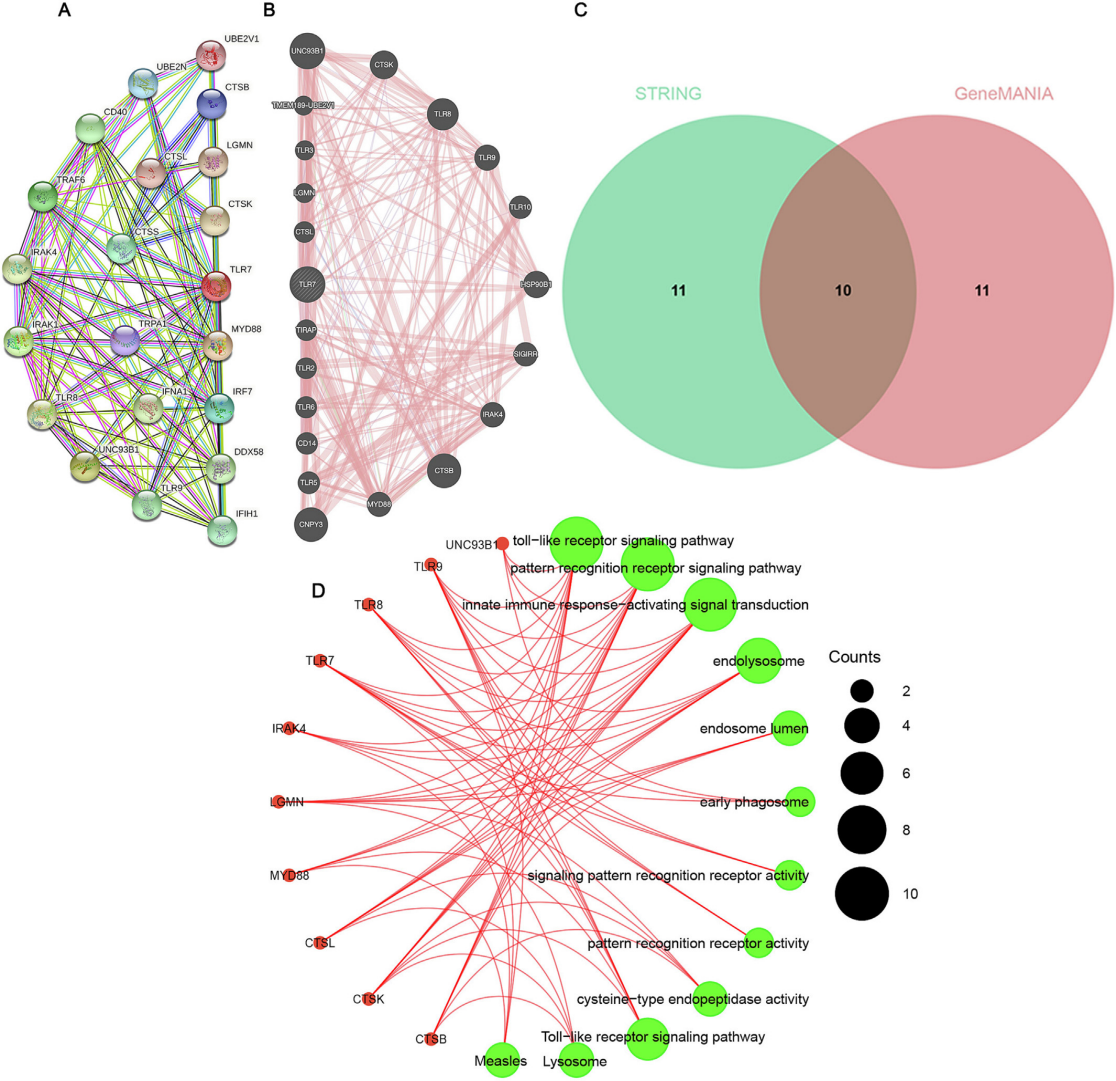
Single-cell analysis of Toll-like receptor 7 (TLR7) expression. A) The detailed expression of TLR7 in 15 cell clusters. B) TLR7 is mainly enriched in Macrophages. C) Venn diagram of intersection of STRING and GeneMANIA. D) Cross-gene enrichment analysis.

As TLR7 was a differential expression in tumor and normal tissue, we further investigated TLR7 expression analysis in a single cell. To obtain the detailed expression of TLR7 in single cells, we performed single-cell RNA-sequencing analysis using the PanglaoDB dataset (Fig. 3A). In the lung tissue, we found that the cells were divided into 15 cell clusters, including Alveolar macrophages, B cells, Clara cells, Dendritic cells, Endothelial cells, Ependymal cells, Erythroid-like and erythroid precursor cells, Fibroblasts, Macrophages, Neutrophils, NK cells, Pericytes, Pulmonary alveolar type II cells, T memory cells, and Unknown. Then the filtered TLR7 gene was mainly enriched in Macrophages (Fig. 3B), indicating that TLR7 played an essential role in the immune process.

### 3.4 Gene-gene interaction network construction and function enrichment of TLR7-associated genes

**Fig. 4:**
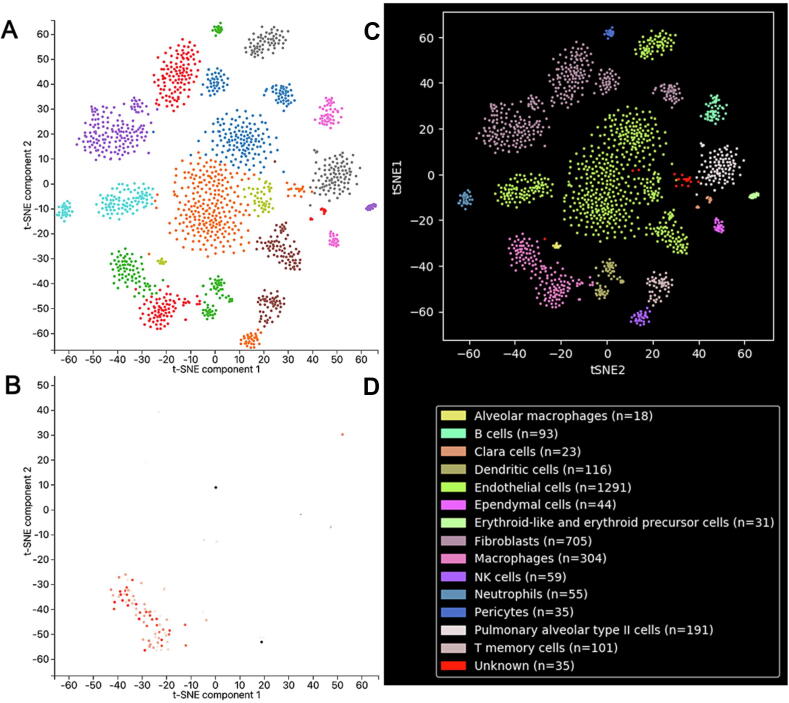
Gene-gene interaction network construction and function enrichment of Toll-like receptor 7 (TLR7) associated genes. A) TLR7-associated protein network mapped using STRING. B) TLR7-related gene network mapped using GeneMANIA. C) Venn diagram of the intersection of STRING and GeneMANIA. D) Cross-gene enrichment analysis.

To understand TLR7-related networks, we used protein–protein, gene-gene interaction networks generated using STRING and GeneMANIA to show that 20 potential target genes and 20 potential target proteins interact with TLR7 (Fig. 4A and 4B). We obtained ten intersecting genes from the intersection of the two datasets and performed GO and KEGG analysis on the ten genes (Fig. 4C). BP was enriched in the toll-like receptor signaling pathway, pattern recognition receptor signaling pathway, and innate immune response-activating signal transduction. CC was mainly enriched in endolysosome, endosome lumen, and early phagosome. Classification analysis revealed that MF was significantly enriched in signaling pattern recognition receptor activity, pattern recognition receptor activity, and cysteine-type endopeptidase activity. KEGG analysis showed significant enrichment of many related pathways, including the Toll-like receptor signaling pathway, Lysosome, and Measles. Subsequently,gene-disease network interaction analysis showed that TLR7 was mainly associated with infectious or post-infectious disorders, immune system disease, cancer or benign tumor, integral system disease, etcetera (Fig. 4D).

### 3.5 The methylation levels of CpG sites in TLR7 promoter

**Fig. 5:**
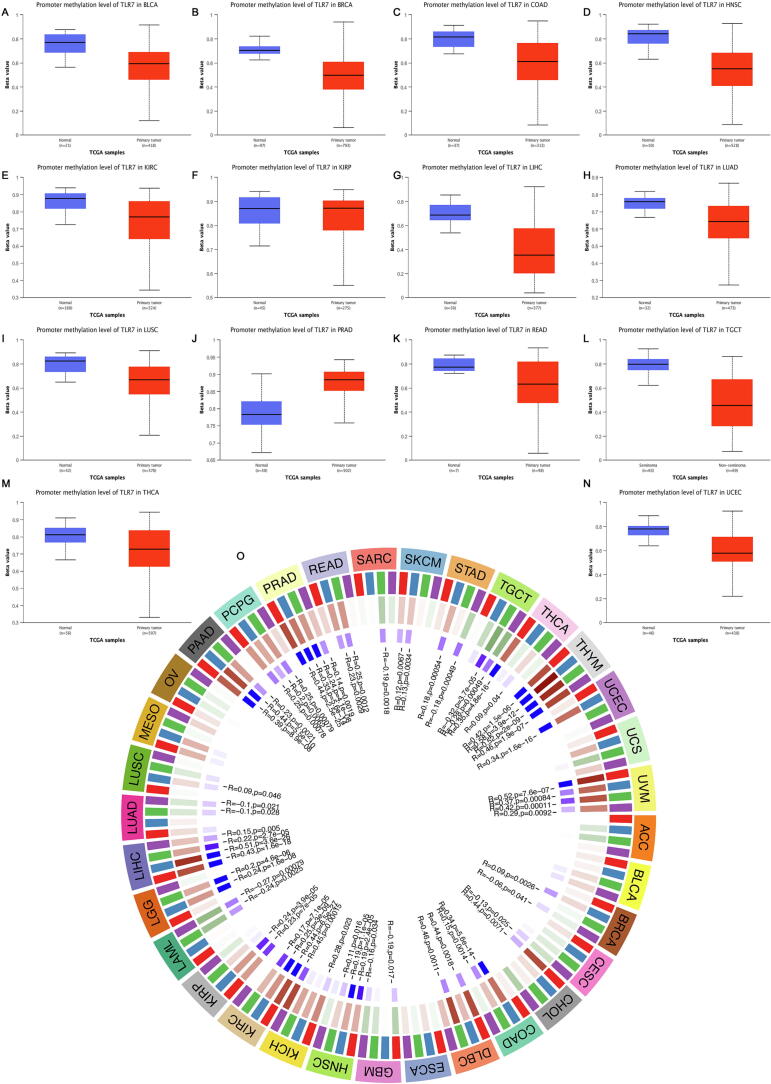
Correlation analysis of TLR7 methylation level and methyltransferase expression level in pan-cancer tissues. A-N) Differential TLR7 methylation levels (β values) between tumors and adjacent normal tissues in the TCGA database. O) Correlation analysis of TLR7 expression with the expression of four methyltransferases. Red represented DNMT1, blue represented DNMT2, green represented DNMT3a, and purple represented DNMT3b. Abbreviations: TLR7, Toll-like receptor 7; TCGA, The Cancer Genome Atlas.

TLR7 was hypermethylated in PRAD, according to promoter methylation analysis. Simultaneously, it was hypomethylated in various cancer types (Fig. 5A). Furthermore, TLR7 methylation appeared to be inversely related to mRNA expression levels in various cancers (*P* < 0.05).

It was well known that DNA methylation was the action of DNA methyltransferases, which covalently bind a methyl group at the 5′ carbon position of cytosines of genomic CpG dinucleotides. However, as shown in Figure 5B, a visual analysis of the relationship between TLR7 expression and the expression of the four methyltransferases revealed that TLR7 and methyltransferase expression levels were primarily significantly positively correlated in all tumors. This finding implies TLR7 may mediate tumorigenesis and progression by regulating epigenetic states.

### 3.6 Genetic variation analysis of TLR7 in pan-cancer

**Fig. 6:**
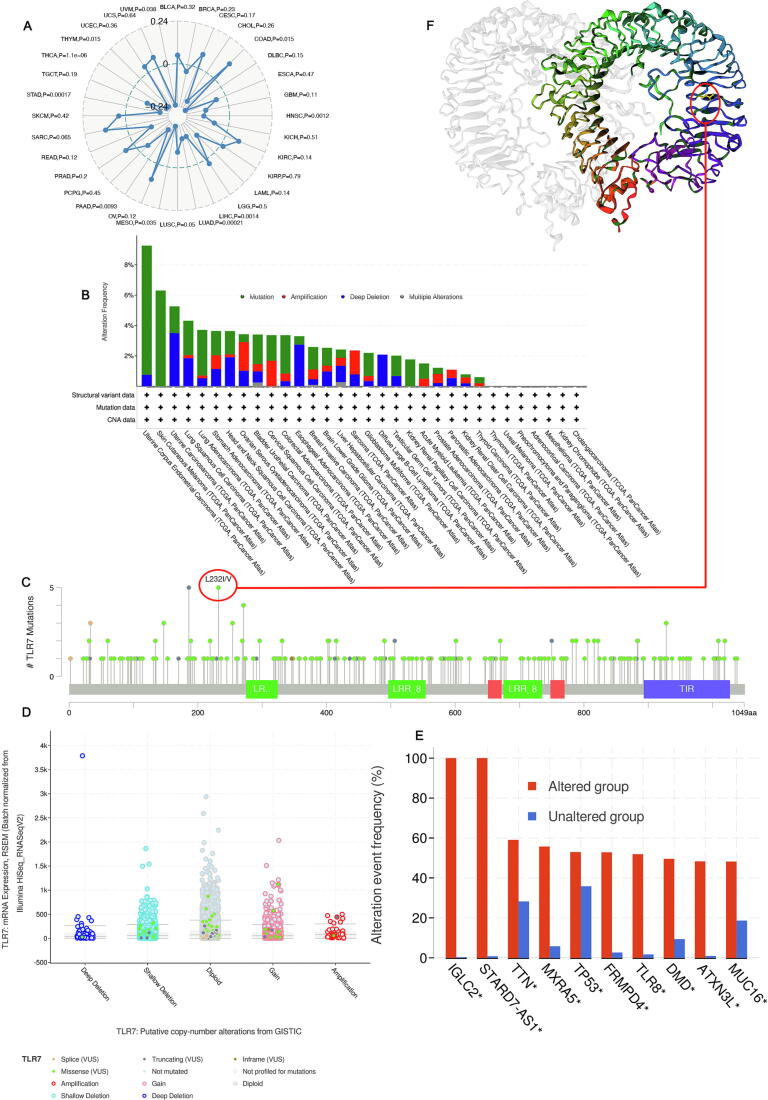
Enetic alterations of TLR7. A) The correlation between TLR7 expression and TMB in pan-cancer was described using Spearman's rank correlation coefficient. B) Summary of changes in TLR7 in the TCGA pan-cancer dataset. C) The type, number, and location of mutations in TLR7 gene alterations. D) Types of TLR7 alterations in pan-cancer. E) Alteration frequencies of related genes in the TLR7-altered and unaltered groups. F) 3D structure of TLR7 at the 232 mutation sites. Abbreviations: TLR7, Toll-like receptor 7; TMB, tumor mutational burden; TCGA, The Cancer Genome Atlas.

TMB was typically expressed as the total number of non-synonymous mutations within an average of 1 M bases in the tumor cell genome coding region, but it was also sometimes expressed directly as the total number of somatic mutations. Base substitution, frameshift mutation, deletion mutation, insertion mutation, and other mutation types were the most common. As a quantifiable indicator, TMB reflected the number of mutations in tumor cells. Correlations between TMB and TLR7 expression in tumor types were analyzed by Spearman. As shown in Fig. 6A, the expression of TLR7 was positively correlated with COAD and negatively correlated with HNSC, LIHC, LUAD, MESO, PAAD, STAD, THCA, THYM, and UVM.

Based on the cBioPortal database, we obtained TLR7 gene alterations in pan-cancer patients with higher mutations in endometrial cancer of the uterus, skin and skin melanoma, uterine carcinosarcoma, lung squamous cell carcinoma, and lung adenocarcinoma.

Mutation (>1.75%) was the primary type (Fig. 6B). The type, site and the number of cases of TLR7 gene modification was further demonstrated (Fig. 6C). TLR7 Missense was the primary type of change, while L232I/V changes were detected in 5 patients. TLR7 gene,

alterations at position 232 of the TLR7 protein, and subsequently observed at position 232 in the 3D structure of the TLR7 protein (Fig. 6F). The most common Diploid, Gain, and Shallow Deletion. (Fig. 6D). IGLC2*, STARD7-AS1*, TTN*, MXRA5*, TP53*, FRMPD4*, TLR8*, DMD*, ATXN3L*, MUC16* gene alterations were more frequent in the altered group than in the unaltered group (Fig. 6E).

### 3.7 TLR7 affects DNA mismatch repair genes and microsatellite instability in pan-cancer

**Fig. 7:**
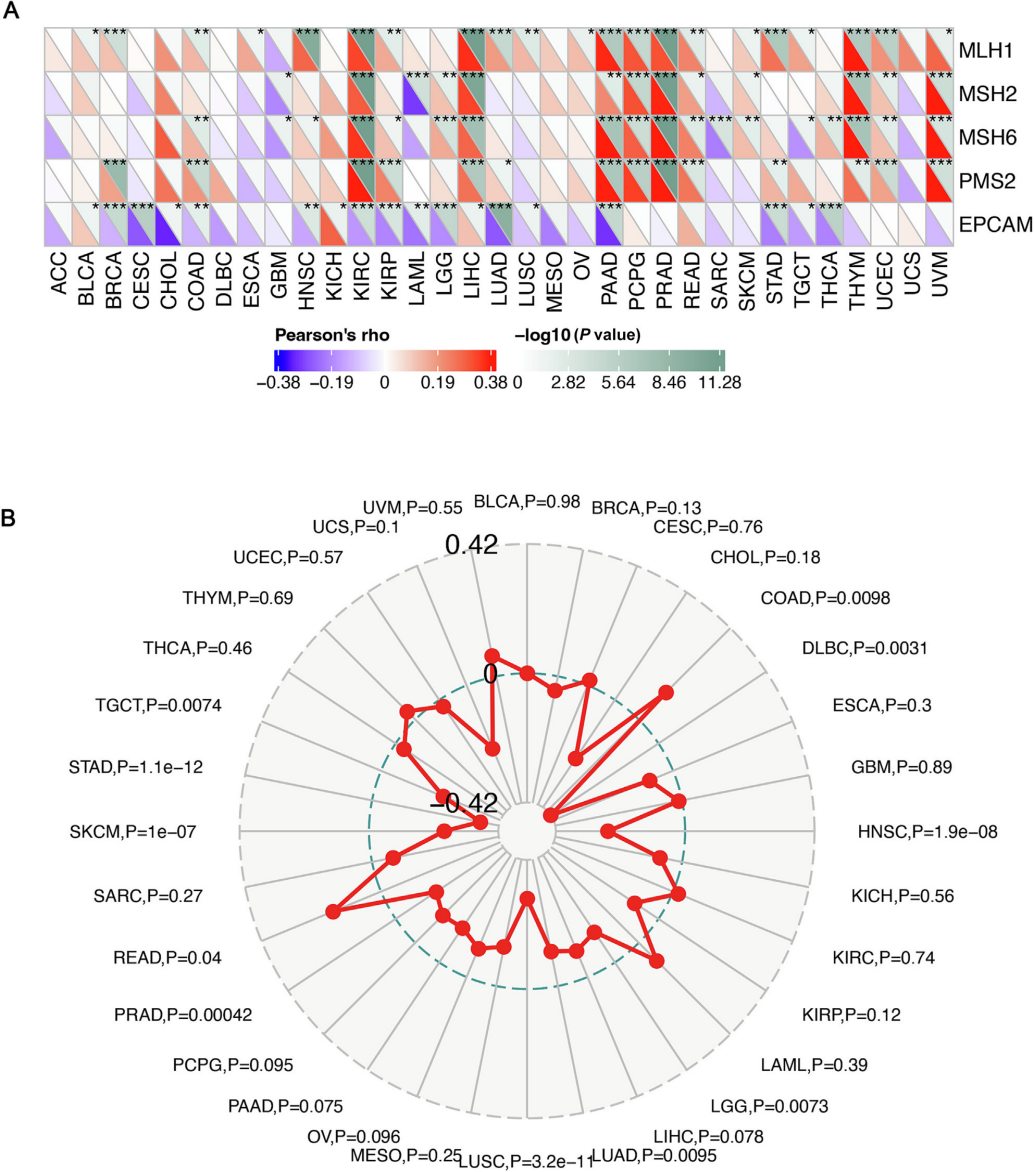
Correlation analysis of TLR7 expression with DNA repair genes and MSI in pan-cancer tissues. A) Correlation analysis of gene expression levels of five MMRs with TLR7 expression. B) Correlation analysis of TLR7 expression and MSI in pan-cancer.Abbreviations: TLR7, Toll-like receptor 7; MMR, mismatch repair; MSI, microsatellite instability.

As shown in Fig. 7A, in KIRC, LIHC, PAAD, PCPG, PRAD, THYM, and UVM, the expression levels of MLH1, MSH2, MSH6, and PMS2 were positively correlated with TLR7, suggesting that in these tumors, TLR7 can upregulate DNA Mismatch repair-related genes to maintain tumor cell viability. Interestingly, we found that EPCAM was inversely associated with most cancers.

Compared with normal tissue, Microsatellite Instability (MSI) is a phenomenon in which new microsatellite alleles appear at a specific microsatellite locus in tumors due to the insertion or deletion of repeat units. The correlation of TLR7 expression with MSI was examined using Spearman's rank correlation coefficient, as shown in Fig. 7B. TLR7 expression in DLBC, HNSC, LGG, LUAD, LUSC, PRAD, SKCM, STAD, and TGCT was significantly negatively correlated with MSI and positively correlated with COAD and READ.

### 3.8 TLR7 associated with immune infiltrating cells

**Fig. 8:**
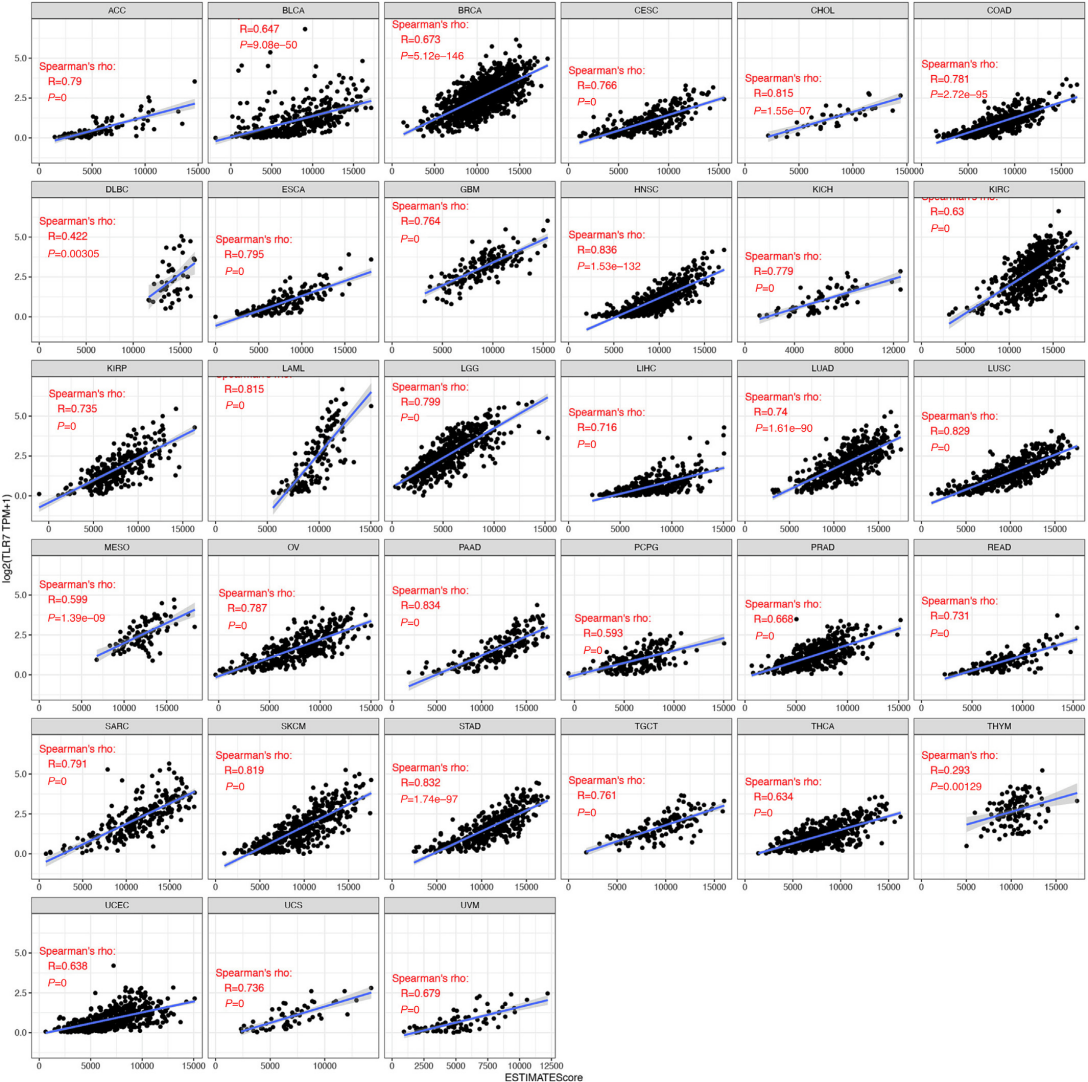
Correlation between Toll-like receptor 7 (TLR7) expression and ESTIMATE score in ACC, BLCA, BRCA, CESC, CHOL, COAD, DLBC, ESCA, GBM, HNSC, KICH, KIRC, KIRP, LAML, LGG, LIHC, LUAD, LUSC, MESO, OV, PAAD, PCPG, PRAD, READ, SARC, SKCM, STAD, TGCT, THCA, THYM, UCEC, UCS, and UVM. Abbreviations: ACCA, drenocortical carcinoma; BLCA, Bladder Urothelial Carcinoma; BRCA, Breast invasive carcinoma; CESC, Cervical squamous cell carcinoma and endocervical adenocarcinoma; CHOL, Cholangiocarcinoma; CHOL, Cholangiocarcinoma; COAD, Colon adenocarcinoma; DLBC, Lymphoid neoplasm diffuse large B-cell lymphoma; ESCA, Esophageal carcinoma; GBM, Glioblastoma multiforme; HNSC, Head and neck squamous cell carcinoma; KICH, Kidney chromophobe; KIRC, Kidney renal clear cell carcinoma; KIRP, Kidney renal papillary cell carcinoma; LAML, Acute myeloid leukemia; LGG, Brain lower grade glioma; LIHC, Liver hepatocellular carcinoma; LUAD, Lung adenocarcinoma; LUSC, Lung squamous cell carcinoma; PRAD, Prostate adenocarcinoma; READ, Rectum adenocarcinoma; STAD, Stomach adenocarcinoma; THCA, Thyroid carcinoma; UCEC, Uterine corpus endometrial carcinoma; UCS, Uterine carcinosarcoma; UVM, Uveal melanoma.

Analysis and visualization of the correlation between TLR7 and immune score (Fig. 8). As shown, nearly all 33 cancers had a significant positive correlation with TLR7 in the ESTIMATE score. In these cancers, the lower the expression of TLR7, the higher the purity of the tumor cells.

### 3.9 The correlation of TLR7 expression with immune checkpoints and immune neoantigens among cancers

**Fig. 9:**
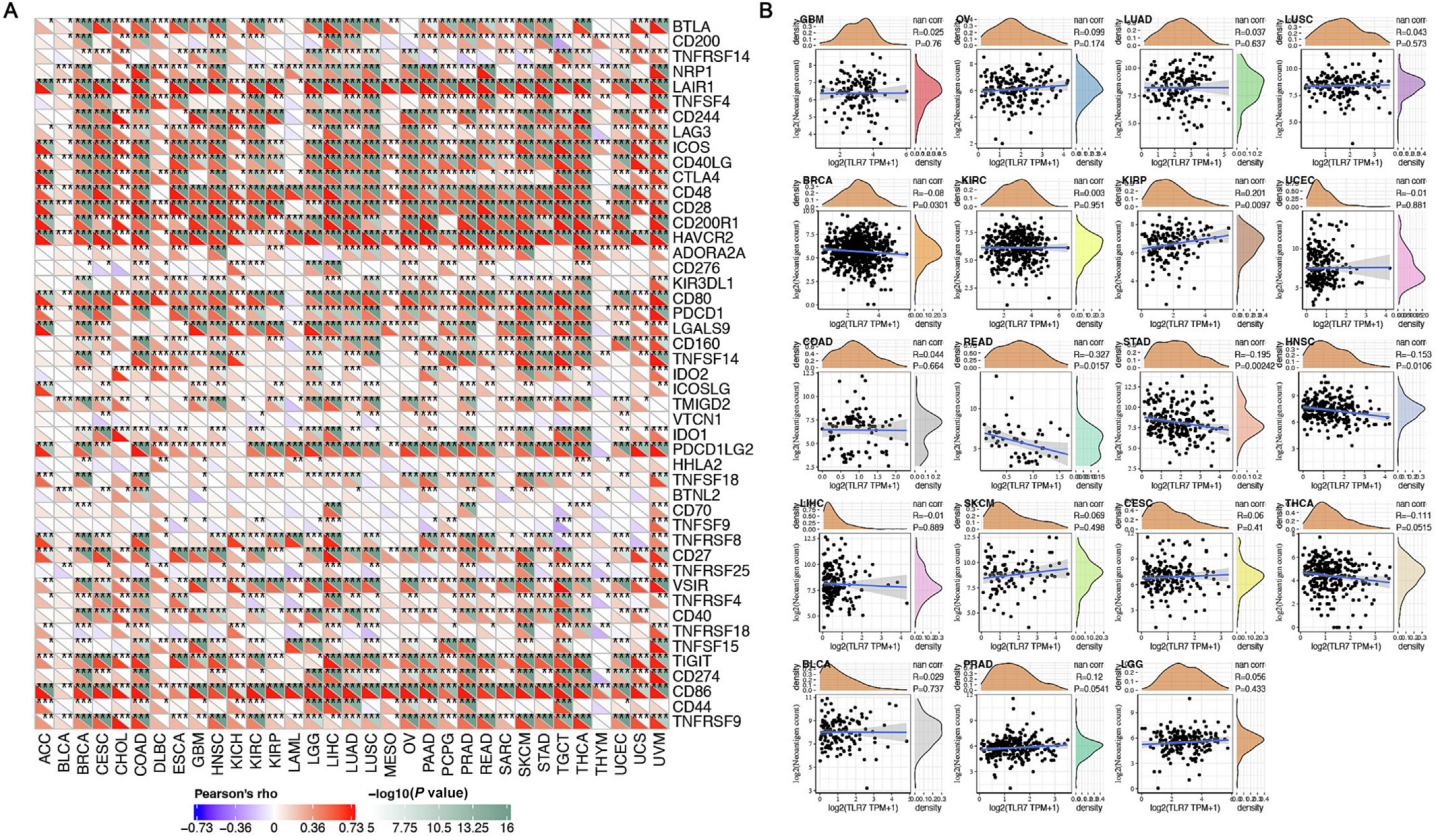
Pan-cancer correlation analysis of Toll-like receptor 7 (TLR7) expression and immune signature. A) Relationship between TLR7 expression and immune checkpoint genes. *P < 0.05, **P < 0.01, **P < 0.001. B) Correlation of TLR7 expression with the number of neoantigens in different types of cancer.

Immune checkpoints were thought to be a group of molecules expressed in immune cells that regulated immune activation and were crucial for controlling autoimmune diseases [27]. Therefore, the relationship between TLR7 expression and immune checkpoint gene expression could be investigated, as shown in Fig. 9A, using the expression data of more than 40 immune checkpoint genes frequently found in different tumors. The findings suggested that TLR7 may have an immunoregulatory function because it was found that the expression of TLR7 was positively correlated with the levels of immune checkpoint gene expression in COAD, LIHC, PRAD, SKCM, STAD, TGCT, UVM, and other tumors, which could affect immune function. We could adjust the expression levels of these immune checkpoint genes by adjusting the expression levels of these immune checkpoint genes,

By taking advantage of the immune activity of tumor neoantigens, mutated genes in tumor cells encode neoantigens, which were referred to as cancer neoantigens, and promote the creation of neoantigen vaccines. We showed that TLR7 expression was positively correlated with the number of neoantigens in KIRP and negatively correlated with BRCA, READ, STAD, and HNSC after counting the number of neoantigens for each cancer type (Fig. 9B).

### 3.10 Pan-cancer analysis of the diagnostic and prognostic value of TLR7 expression

**Fig. 10:**
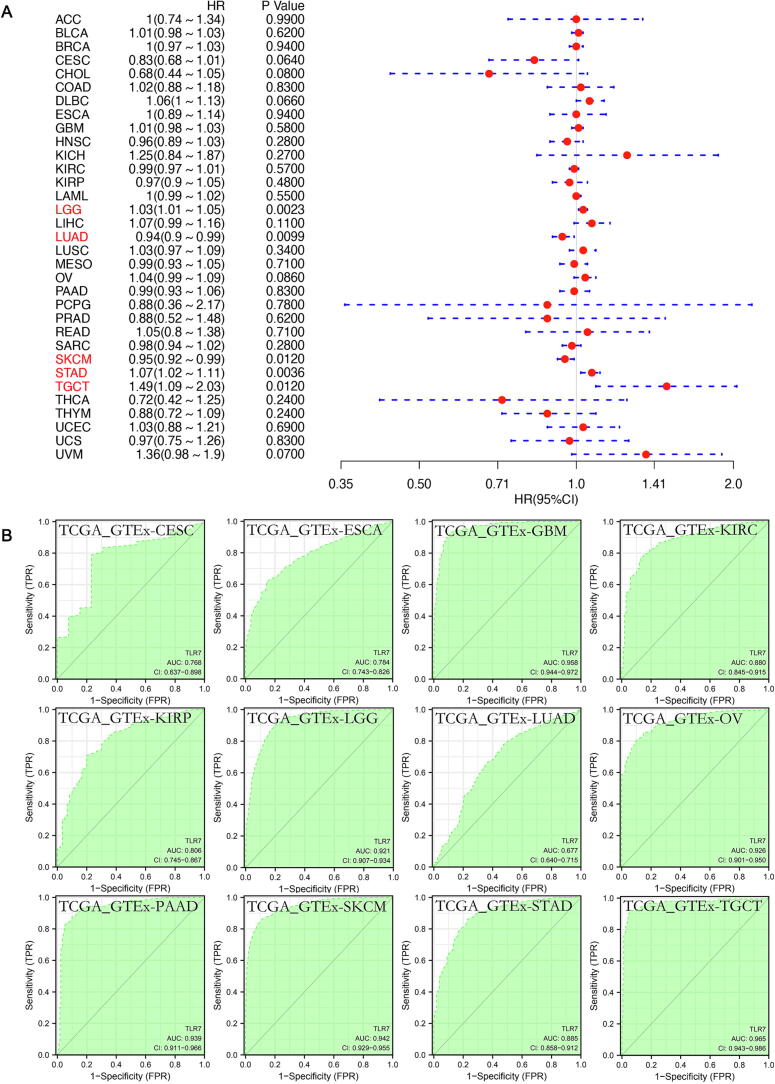
Pan-cancer analysis of the diagnostic, prognostic value of Toll-like receptor 7 (TLR7) expression. A) Forest plot showing hazards regression model (HR) and 95% confidence interval (CI) for TLR7 expression associated with cancer Overall survival (OS). B) Receiver operating characteristic (ROC) curves of TLR7 in 12 cancers.

Next, univariate Cox regression was used to examine the relationship between TLR7 expression and cancer prognosis. Based on the levels of TLR7 expression, two subgroups of cancer cases were identified. TLR7 expression had a significant impact on OS in patients with LGG (HR = 0.03, *P* = 0.0023), LUAD (HR = 0.94, *P* = 0.0099), SKCM (HR = 0.95, *P* = 0.0120), STAD (HR = 1.07, *P* = 0.0035), and TGCT (HR = 1.49, *P* = 0.0120), according to a forest plot of 33 tumors (Fig. 10A). In Fig. 10B, AUC had sensitivity and specificity and was often used to indicate the intrinsic validity of a diagnostic test [28], [29], [30]. The results showed that TLR7 had a excellent diagnostic value for a variety of cancers, including CESC (AUC = 0.768), ESCA (AUC = 0.784), GBM (AUC = 0.958), KIRC (AUC = 0.880), KIRP (AUC = 0.806), LGG (AUC = 0.921), LUAD (AUC = 0.677), OV (AUC = 0.926), PAAD (AUC = 0.939), SKCM (AUC = 0.942), STAD (AUC = 0.885), TGCT (AUC = 0.965).

### 3.11 TLR7 drug sensitivity analysis

**Fig. 11:**
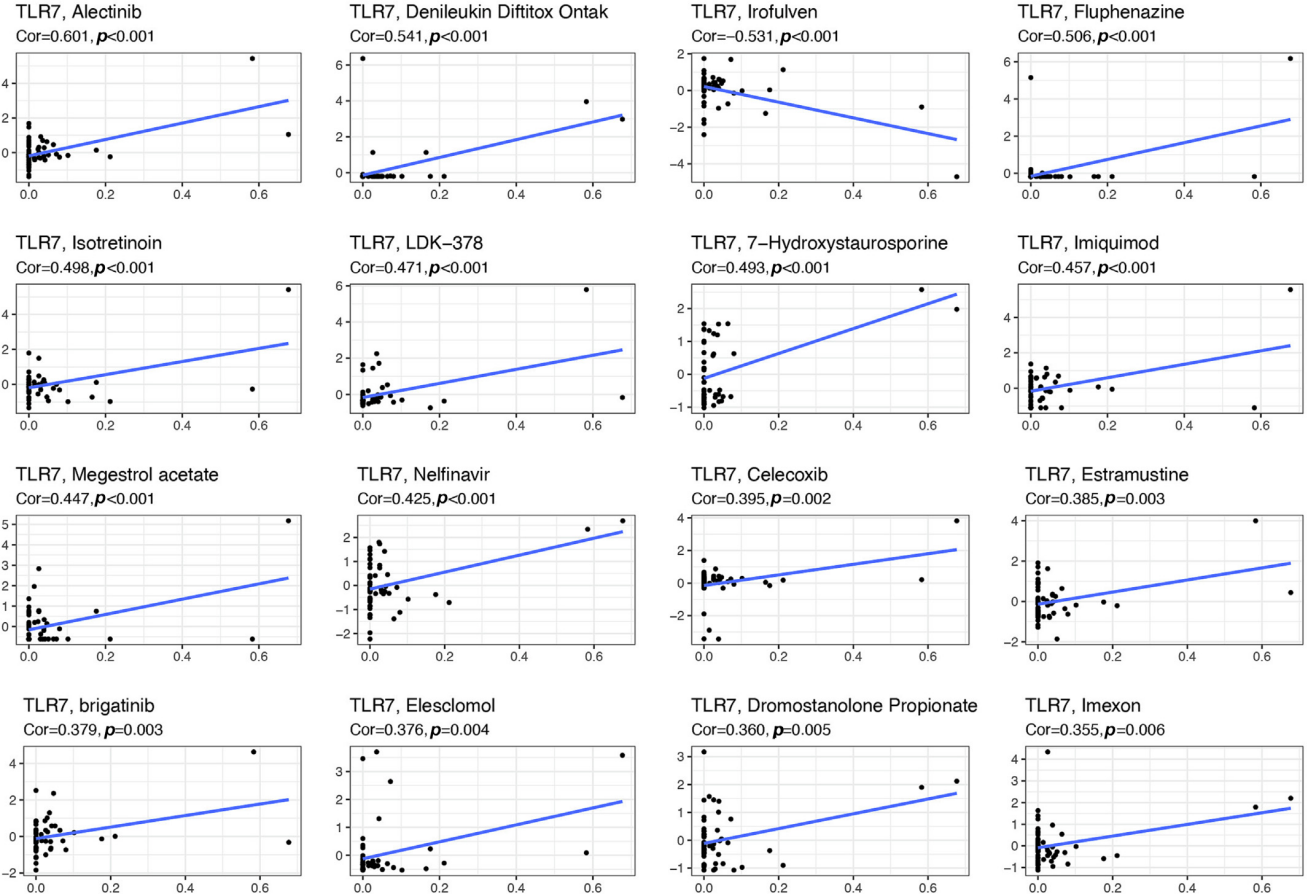
Drug sensitivity analysis of Toll-like receptor (TLR7).

To investigate the relationship between TLR7 expression and drugs, we further analyzed through the CellMiner™ database (Fig. 11). Notably, TLR7 expression was inversely correlated with the Irofulven sensitivity. In addition, our results showed that TLR7 expression was positively correlated with the sensitivity of Alectinib, Denileukin Diftitox Ontak, Fluphenazine, Isotretinoin, LDK − 378, 7 − Hydroxystaurosporine, Imiquimod, Megestrol acetate, Nelfinavir, Celecoxib, Estramustine, brigatinib, Elesclomol, Dromostanolone Propionate, and Imexon.

## 4 Discussion

Since December 2019, COVID-19 has caused a global pandemic and has become a serious global public health problem [31], [32]. In addition, studies have found that cancer patients are very susceptible to SARS-CoV-2 infection, which affects their prognosis [33], [34]. Nevertheless, a standardized analytical framework to define aberrant gene expression and pathogenesis in human cancers did not yet exist.

TLR7 is a gene that codes for proteins. Innate and adaptive immunity were aided by endosomal receptors [35], [36]. In addition, they controlled host immune reactions to pathogens by identifying guanosine analogs or single-stranded RNA (ssRNA) derived from viruses [31], [37], [38], [39]. The TIR domains of the two molecules came into direct contact during dimerization after binding to the agonist, which attracted the TIR-containing downstream linker MYD88[38] through homotypic interactions. In turn, activation of the downstream transcription factor NF-κB caused the secretion of proinflammatory cytokines and interferon due to the formation of a Myddosome signaling complex involving IRAK4, IRAK1, TRAF6, and TRAF3 [31], [38].

This study used multiple databases to analyze changes in TLR7 mRNA in tumors. Furthermore, analysis was done on the relationship between immune infiltration, TLR7 expression levels, and the prognosis of these tumors. In addition, we discussed the potential prognosis of patients following SARS-CoV-2 infection in the various tumors.

Findings from disease network analysis TLR7 were linked to immune system disorders, cancer or benign tumors, infectious or post-infectious disorders, and integumentary system diseases, reflecting its widespread distribution and essential function in the human body. Furthermore, abnormal DNA methylation is highly correlated with tumors' development, growth, and cell carcinogenesis [40]. Cancer genes were significantly hypomethylated compared to their regular counterparts [41]. Again, our study's results were consistent, suggesting that TLR7 may affect DNA methylation and encourage the development of tumors, though the exact mechanism was still unknown. High homology exists between SARS-CoV and SARS-CoV-2 [42]. Therefore, we used the GSE52920 database to study the TLR7 alterations in SARS-CoV-infected mice. The results showed that TLR7 expression levels increased following SARS-CoV infection. This finding suggested TLR7 levels might rise in tumor tissue following SARS-CoV-2 infection.

The survival environment for tumor cells to proliferate and metastasize in deep tissues was the tumor microenvironment, which was composed of tumor cells, immune cells, stromal cells, and a variety of active molecules [43]. This study discovered a strong positive correlation between TLR7 expression and nearly every tumor ESTIMATE score. Recently, there had some evidence that immune-related TLR7 expression was related to cancer [44]. Several TLR7 functions were involved in regulating the tumor immunology of specific tumors, as investigated in the present study.

More than 40 checkpoints were correlated with TLR7 expression levels in different tumors, and it was positively correlated with COAD, LIHC, PRAD, SKCM, STAD, TGCT, and UVM. In addition, immune checkpoint molecules were upregulated with TLR7 overexpression, allowing these tumor cells to evade immune surveillance. According to the data, TLR7 expression may impact how cancer patients react to immune checkpoint therapy, which would help us understand how immunotherapy works to treat cancer better.

It is critical to developing or repurposing novel cancer drugs [45], [46], [47]. In addition, we discovered that TLR7 expression correlates with sensitivity to many drugs, including Alectinib, Denileukin Diftitox Ontak, and Fluphenazine, using the CellMinerTM database. Thus, we concluded that TLR7 might be involved in chemotherapy and possibly have something to do with chemotherapy resistance.

We knew the new coronavirus was an RNA virus, and RNA viruses generally had a high mutation frequency during replication. Another current research direction of TLR7 recognition of viruses is that this recognition allows viruses to adjust their genome composition during evolution to evade this recognition. In addition, some inactivated single-stranded RNA viruses cannot activate immune responses, and viruses evade TLR7 recognition. Therefore, our research on the correlation between the new coronavirus and TLR7 had great clinical significance [48]. The GEO dataset results showed that the TLR7 detected in samples of mice infected with COVID-19 increased, which was consistent with the response of cytoplasmic nucleic acid receptors to recognize intracellular damage caused by exogenous pathogen infection. Although the rapid induction of type I interferon-induced limited the propagation of the virus, persistent increases in type I interferon levels late in infection were associated with abnormal inflammation, such as severe lung inflammation, and may also exacerbate the poor prognosis of cancer patients with COVID-19.

However, our study still had some shortcomings,. Firstly, this study had no actual experimental or clinical data; instead, the conclusions were solely based on bioinformatics analysis. In addition, cellular-level analysis of immune cell markers may introduce systematic bias because tumor tissue information was primarily derived from massive microarray and sequencing data from public databases. Thirdly, we could not establish a direct causal link, although we discovered a correlation between TLR7 expression and patient survival in some tumor patients and that TLR7 altered immune cell infiltration. Future biological research was required to elucidate and confirm the role of TLR7 in cancer.

In summary, TLR7 expression levels were significantly abnormal in different cancer types. As an immune-related biomarker, TLR7 can diagnose and predict the prognosis of cancer patients with COVID-19 and is a potential therapeutic target for these patients.

## 5 Conclusion

TLR7 expression, survival prognosis, gene mutation, MMR, MSI, TMB, tumor immune microenvironment, functional pathways, and drug sensitivity were all demonstrated in a pan-cancer analysis. Therefore, TLR7 was expected to be a potential target for COVID-19 cancer therapy based on its abnormal expression in pan-cancer and the significant differences in prognosis and immune environment. This study highlighted TLR7’s multifaceted role in pan-cancer and provided new insights into TLR7’s potential role in drug regulation.
